# Paracingulate Sulcus Morphology and Hallucinations in Clinical and Nonclinical Groups

**DOI:** 10.1093/schbul/sby157

**Published:** 2018-10-30

**Authors:** Jane R Garrison, Charles Fernyhough, Simon McCarthy-Jones, Jon S Simons, Iris E C Sommer

**Affiliations:** 1 Department of Psychology, University of Cambridge, Cambridge, UK; 2 Behavioural and Clinical Neuroscience Institute, University of Cambridge, Cambridge, UK; 3 Department of Psychology, Durham University, Durham, UK; 4 Department of Psychiatry, Trinity College Dublin, Dublin, Ireland; 5 Department of Neuroscience, Rijks Universiteit Groningen (RUG), University Medical Center Groningen, Groningen, Netherlands; 6 Department of Medical and Biological Psychology, University of Bergen, Bergen, Norway

**Keywords:** hallucinations, paracinguate sulcus, clinical, nonclinical

## Abstract

Hallucinations are a characteristic symptom of psychotic mental health conditions that are also experienced by many individuals without a clinical diagnosis. Hallucinations in schizophrenia have been linked to differences in the length of the paracingulate sulcus (PCS), a structure in the medial prefrontal cortex which has previously been associated with the ability to differentiate perceived and imagined information. We investigated whether this putative morphological basis for hallucinations extends to individuals without a clinical diagnosis, by examining whether nonclinical individuals with hallucinations have shorter PCS than nonclinical individuals without hallucinations. Structural MRI scans were examined from 3 demographically matched groups of individuals: 50 patients with psychotic diagnoses who experienced auditory verbal hallucinations (AVHs), 50 nonclinical individuals with AVHs, and 50 healthy control subjects with no life-time history of hallucinations. Results were verified using automated data-driven gyrification analyses. Patients with hallucinations had shorter PCS than both healthy controls and nonclinical individuals with hallucinations, with no difference between nonclinical individuals with hallucinations and healthy controls. These findings suggest that the association of shorter PCS length with hallucinations is specific to patients with a psychotic disorder. This presents challenges for full-continuum models of psychosis and suggests possible differences in the mechanisms underlying hallucinations in clinical and nonclinical groups.

## Introduction

Hallucinations are a common and debilitating symptom associated with several mental health disorders, but are also experienced by many individuals without a clinical disorder. Questions remain over the extent to which the mechanisms underlying hallucinations in clinical and nonclinical groups are the same, with those related to clinical diagnoses lying at one extreme of a continuum of experience.^1,2^ Such a continuum might be fully continuous if hallucinations arise from a single factor or process, or nearly fully continuous if there are very large number of relevant factors. Alternatively, there may be a quasi-dimensional continuum if hallucinations arise from the interaction of a small number of relevant factors.^3^ Such a quasi-dimensional model would be consistent with observations of discontinuity in the experience of hallucinations within the general population,^4^ and thus with possible differences in the underlying neural processes that might explain the variability in the subjective experience of hallucinations between clinical and nonclinical groups.

**Fig. 1.**
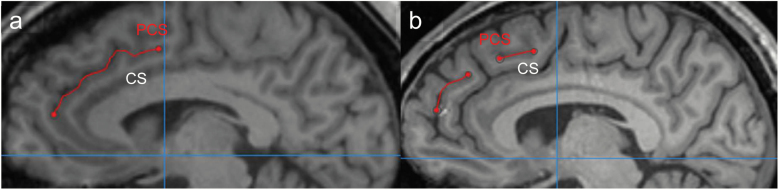
Paracingulate sulcus (PCS) measurement for 2 example images. *Note:* The PCS lies dorsal and parallel to the cingulate sulcus (CS). (a) The PCS is continuous and is measured from its origin in the first quadrant (cross-hairs at *y* = 0 and *z* = 0) to its end. (b) The PCS is noncontinuous, and is measured from its start in the first quadrant with subsequent segments included such that the total distance between them is less than 20 mm.

Hallucinations in patients diagnosed with schizophrenia are often associated with impairment in reality monitoring, the cognitive ability to distinguish between real and imagined information.^5,6^ With neuroimaging studies of reality monitoring in healthy individuals repeatedly revealing activity within the anterior medial prefrontal cortex (mPFC),^7–9^ recent reality monitoring research has focused on the paracingulate sulcus (PCS), a structure that lies in the dorsal anterior cingulate region of the mPFC (figure 1). Among the last sulci to develop in utero, the PCS shows significant inter-individual variation, being completely absent in 12%–27% of brain hemispheres measured in healthy individuals.^10–12^ Healthy individuals with no discernable PCS in either brain hemisphere show reduced reality monitoring accuracy compared with individuals with a visible PCS in one or both hemispheres of the brain.^13^ With paracingulate folding known to be reduced in patients with schizophrenia (eg, Park et al^14^), recent research which investigated PCS morphology in patients who were distinguished by whether they experienced hallucinations, revealed that hallucinations were associated with a significant reduction in PCS length.^15^ This suggests a specific morphological basis for these experiences within the PCS, which might be associated with an impairment in reality monitoring contributing to the attribution failure underlying hallucinations in schizophrenia.

A fundamental proposition of the theoretical source monitoring framework of Johnson and Raye^5^ is that misidentification of the origin of information can arise from a number of factors (eg, vivid perceptual imagery, differences in evaluative criteria, or cognitive contextual features). Different factors may thus be associated with the experience of hallucinations across different groups. As such, research has questioned whether healthy individuals with hallucinations show the reality monitoring impairment observed in experimental tasks and implicated in the hallucinations experienced by people diagnosed with schizophrenia. While a meta-analysis^16^ reported differences in reality monitoring ability between hallucination-prone and nonprone healthy individuals, this analysis was based on 3 studies, only 2 of which are in the published domain.^17,18^ We ourselves carried out 2 separate reality monitoring studies using verbal tasks, finding no evidence of a link with proneness to auditory verbal hallucinations (AVHs) in the general population.^19^ However, Sugimori et al^20^ found that AVH experience scores in nonclinical participants were correlated with superior temporal gyrus activity for words that participants incorrectly called “heard” but had actually previously imagined, suggesting a link between AVHs and sensory activity in the identification of source. Analogous findings have been reported for the visual modality.^21^

AVHs are the most common modality of hallucinations reported by patients with mental health disorders, but are also experienced in the general population by around 12% of children/adolescents and 6–7% of adults (life-time experience).^22,23^ AVHs include such experiences as auditory imagery, intrusive thoughts, and vivid voices of other people.^24^ While descriptions of hallucinatory experience are similar in individuals with and without clinical diagnoses in terms of localization, loudness, number of voices, and external attribution of the perceptual experience,^25,26^ there are differences in frequency, perception of control, age of onset,^3^ preponderance of male voices,^27^ and more negative content of clinical AVHs.^3,25^ Indeed, a recent systematic review found that only 52% of the 21 features of hallucinations studied were experienced by individuals in both groups,^28^ suggesting the full-continuum model might not be an accurate representation of the variation in hallucinations experienced by individuals with and without a clinical diagnosis.

**Fig. 2.**
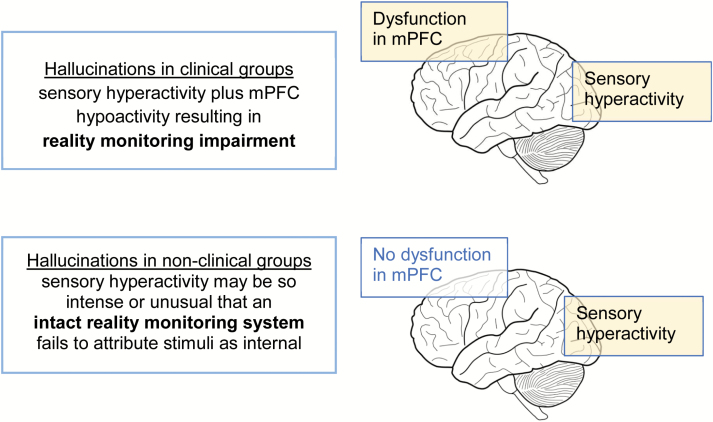
Possible mechanisms underlying hallucinations in clinical and nonclinical groups.

In light of these differences in the experience of AVHs, and consistent with a quasi-continuous model implicating the involvement of several significant underlying factors, we propose that there may be differences in the mechanisms by which hallucinations are generated in clinical and nonclinical groups (figure 2).^6^ We suggest that hyperactivation of sensory cortices, which evidence suggests is associated with the perceptual content of both clinical and nonclinical hallucinations,^29,30^ may be subject to a process of reality monitoring mediated by cortical activity within the dorsal anterior cingulate cortex (ACC) region of the mPFC. In healthy individuals without hallucinations, internally generated sensory activity may be correctly identified by effective reality monitoring processes leading to the correct recognition of the associated perceptual content as self-generated. In individuals with hallucinations, such sensory activity may be more intense, perhaps mediated by stress, trauma, or fatigue.^3^ When accompanied by hypoactivation of mPFC as observed in individuals with a clinical diagnosis,^31^ this may lead to reality monitoring failure to recognize the sensory activity as self-generated, resulting in the experience of a hallucination. In nonclinical individuals with hallucinations, the sensory hyperactivity may be of sufficient intensity, or unusual in character (perhaps in terms of vividness), that an otherwise intact reality monitoring system might fail to recognize the stimuli as internally generated, leading to a hallucination. This proposal is thus consistent with the idea of multiple factors contributing to reality monitoring judgments.^5^ It also develops the earlier neuroanatomical model proposed by Allen et al,^29^ but suggests a varied contribution of the different factors implicated in hallucination generation between individuals with and without a clinical diagnosis. In the case of nonclinical hallucinations, the emphasis is predominantly on the hyper-activation of sensory cortices, whereas for clinical hallucinations, there is an additional effect of impairments in top-down monitoring processes, associated with mPFC dysfunction.

In light of this framework, we were motivated to investigate whether the experience of hallucinations in individuals without a clinical diagnosis shows any association with variable paracingulate sulcal morphology—the failure to detect such variability being consistent with the absence of a behavioral reality monitoring impairment.

Here, we investigate PCS length in both hemispheres of the brain in 3 matched groups: patients with a clinical diagnosis who experience AVHs, individuals with no clinical diagnosis who experience frequent AVHs (at least once a week) but without delusions, and healthy controls, with no life-time experience of hallucinations. PCS length was measured from structural MRI scans using a previously validated measurement protocol carried out blind to group status, with manual tracing morphological findings compared with those obtained using automated measures of local gyrification. It was hypothesized that PCS length would be shorter, and measures of gyrification around the paracingulate cortex smaller, in both clinical and nonclinical individuals with hallucinations compared with nonclinical individuals without hallucinations.

## Methods

### Participants

Fifty nonclinical participants with AVHs, 50 patients with a psychotic disorder and AVHs, and 50 healthy control subjects were matched for age, gender, handedness, and years of education. There were no differences between the groups for intracranial volume (table 1).

**Table 1.** Participant Data

	Healthy Controls	Clinical Hallucinations	Nonclinical Hallucinations	Test Statistic	*P* Value
*N*	50	50	50		
Males (*N*)	22	20	20	χ^2^ = 0.220	.896
Right handed (*N*)	38	42	38	χ^2^ = 1.271	.530
Age (years)	39.2 (14.3)	39.4 (10.8)	43.2 (13.0)	*F*(2, 147) = 1.544	.217
Education (years)	6.5 (2.1)	7.0 (3.0)	6.8 (2.4)	*F*(2, 147) = 0.458	.633
Intracranial volume (mm^3^ × 10^3^)	1422 (276)	1362 (252)	1416 (228)	*F*(2, 147) = 0.843	.433

*Note:* Parentheses = standard deviation; 10 patients received no anti-psychotic medication, 29 atypical anti-psychotics, 10 typical anti-psychotics, 1 both. Limited phenomenology and symptom severity data were available on 40 individuals with nonclinical hallucinations and 29 of the patients. Using PSYRATS (Psychotic Symptoms Rating Scales),^32^ there were significant differences between the clinical and nonclinical groups on measures of hallucination frequency, duration, loudness, degree of negative content, distress amount, and intensity, disruption to daily life and control over voices (χ^2^ > 9.767, *P* < .021). In PSYRATS measures relating to symptom severity, the clinical group scored consistently higher.

Details of the recruitment procedure for participants are given in supplementary material. All patients met criteria for schizophrenia (29 participants), schizoaffective disorder (7), nonspecific psychosis (13), or schizophreniform disorder (1), and all experienced AVHs.

Analysis of gray matter volume (GMV) differences for some of this scan data has previously been undertaken, but no prior analysis of paracingulate morphology has been carried out.

### Imaging Data, Measurement of PCS Length, and Calculation of Local Gyrification Index

Details of the scanning protocol, measurement of PCS length (figure 1),^15^ and calculation of local gyrification indices^33^ are given in supplementary material.

## Results

### PCS Measurement Differences Associated With Hallucinations in Clinical and Nonclinical Groups

There was a significant difference in total PCS length (PCS length summed across both hemispheres) between the 3 matched groups (patients with AVHs, nonclinical individuals with AVHs, and healthy controls), *F*(2, 147) = 11.002, *P* < .001, $\eta_{p}^{2}=.\text{13}0$. This result survived the addition of cortical surface area for each brain scan, as a covariate, *F*(2, 146) = 8.032, *P* < .001, $\eta_{p}^{2}=.099$, thus controlling for a possible effect of brain size. Other potential covariates such as age, intracranial volume, and global brain gyrification index had no significant effect on PCS length and were removed from the model.

**Fig. 3.**
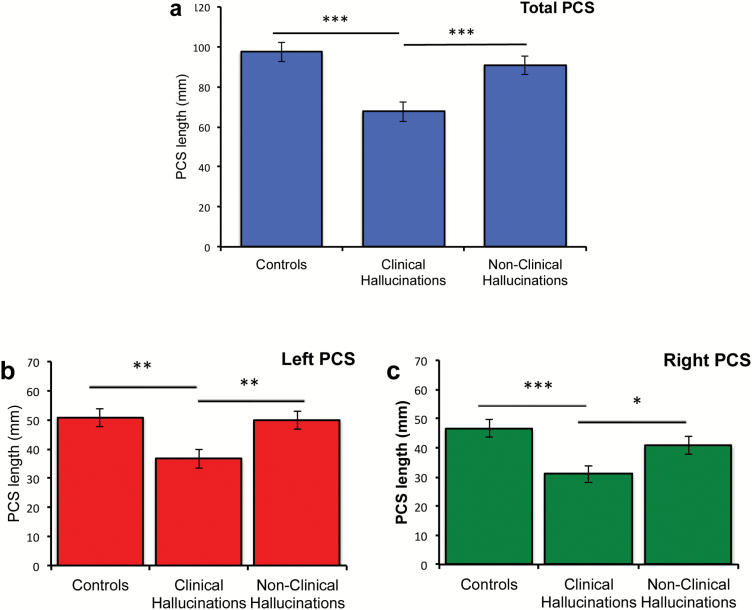
Paracingulate sulcus (PCS) length by group. (a) Total PCS length across both hemispheres, (b) PCS length in the left hemisphere, and (c) PCS length in the right hemisphere. ***P < .001, **P < .01, *P < .05. Error bars represent standard error of the mean. Variation is also seen between the groups for the proportions of absent PCS. Taking an earlier definition of an absent PCS to be one of length <20 mm,^11,12^ 11% of the brain hemispheres measured in control subjects had no PCS compared with 20% for nonclinical individuals with hallucinations, and 33% for individuals in the clinical group.

Planned comparisons revealed that patients with AVHs exhibited significantly reduced PCS length compared with healthy controls, *t*(98) = 4.400, *P* < .001, *d* = .894 (mean reduction = 29.80 mm), as well as with nonclinical individuals with AVHs, *t*(98) = 3.472, *P* = .001, *d* = .694 (mean reduction = 25.15 mm). However, there was no significant reduction in sulcal length in nonclinical individuals with AVHs compared with healthy controls, *t*(98) = 1.013, *P* = .314, *d* = .202 (figure 3). Consistent with earlier findings,^15^ we also found main effects of hemisphere, *F*(1, 147) = 6.946, *P* = .009, $\eta_{p}^{2}=.0\text{45}$, but no interaction between hemisphere and group. PCS length was greater in the left hemisphere than the right hemisphere in all participant groups, *t*(149) = 2.647, *P* = .009, *d* = .272. Patients with AVHs exhibited shorter PCS length compared with healthy controls in both hemispheres, *t*(98) > 3.182, *P* < .002, *d* > .636, and shorter PCS length compared with nonclinical individuals with AVH, in both hemispheres, *t*(98) > 2.245, *P* < .027, *d* > .449. There was no significant difference in PCS length in either hemisphere between healthy control participants and nonclinical individuals with AVHs, *t*(98) < 303, *P* > .196, *d* < .261. There was also no significant association between PSYRATS symptom severity measures and either left or right PCS length for individuals who experience either clinical (data available for *n* = 29) or nonclinical hallucinations (*n* = 40), or when collapsed across both groups (*r* < .219, *P* > .07, uncorrected for multiple comparisons).

### Automated Local Gyrification Analyses

To validate the PCS measurement findings, we conducted separate automated analyses of surface-based lGI (see supplementary methods for an explanation of this approach). A significant reduction in mean gyrification in the mPFC regions of interest surrounding the PCS (bilateral frontopolar, medial orbitofrontal, superior frontal, and paracentral cortices) was found in patients with AVHs when compared with healthy controls, *t*(98) = 2.128, *P* = .036, *d* = 0.425. Significant differences after correcting for multiple comparisons were also detected in the left lateral surface within the pars opercularis, inferior parietal and precentral parcellations, *t*(98) > 3.26, *P* < .001, *d* > 0.725.

**Fig. 4.**
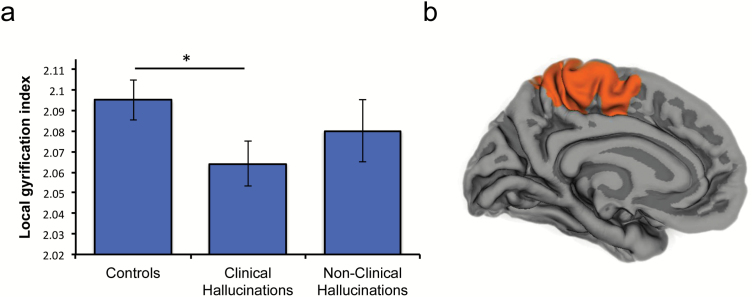
Cortical gyrification differentiates patients with auditory verbal hallucinations (AVHs), but not nonclinical individuals with hallucinations, from healthy controls. (a) Mean lGI in bilateral regions surrounding the paracingulate sulcus (PCS) is lower in patients with hallucinations than in healthy controls, *P < .05, error bars represent standard error of the mean. (b) Local gyrification index around posterior PCS significantly differentiates clinical individuals with AVHs from healthy controls. There were no significant differences in lGI between nonclinical individuals with AVHs and either healthy controls, or patients with hallucinations.

We found no significant differences in mean lGI between nonclinical individuals with AVHs and either healthy controls or patients with hallucinations in these mPFC regions of interest *t*(98) < .846, *P* > .400, *d* < 0.169 (figure 4), and no further differences across the rest of the brain that survived correction for multiple comparisons. These parcellation findings were confirmed by a whole brain analysis using a Monte Carlo procedure for multiple comparison correction. Differences in lGI were revealed in the PCS for the contrast of patients with AVHs compared with healthy controls. There were no significant clusters anywhere across the brain for the contrast of nonclinical individuals with AVHs with healthy controls, or with patients with AVHs.

## Discussion

We used a previously validated visual classification technique and automated data-driven analysis to demonstrate that patients with schizophrenia who hallucinate exhibit reduced PCS length compared with both healthy controls and individuals who hallucinate in and have no clinical diagnosis. There was no difference between the hallucinating and nonhallucinating groups in terms of age, sex, handedness, years of education, and brain volume. Nonclinical individuals with hallucinations had longer PCS length in both hemispheres of the brain compared with patients with hallucinations, but showed no significant difference when compared with healthy control subjects. We verified these results using a data-driven gyrification analysis, finding differences in lGI in regions surrounding the PCS between patients with hallucinations and healthy controls, but not between nonclinical individuals with hallucinations and either healthy controls or patients with hallucinations. Together, these findings suggest that the association of shorter PCS length with hallucinations is specific to patients with a psychotic disorder.

These results are in line with previous findings that the reality monitoring impairments associated with hallucinations in schizophrenia may not extend to nonclinical individuals with hallucinations.^19^ The findings are thus consistent with the framework outlined in the Introduction section which proposes differences in the mechanisms underlying hallucinations in clinical and nonclinical groups (figure 2). This framework suggests that nonclinical hallucinations are predominantly associated with sensory hyperactivity, without the impairment in the reality monitoring process and morphological differences in paracingulate cortex associated with hallucinations in patients with schizophrenia.

Further evidence for the proposed framework comes from the failure to detect dopaminergic dysfunction in nonclinical individuals with hallucinations.^34^ Increased dopamine synthesis has been associated with the development of psychosis (eg, Howes et al^34,35^), and suggested to relate to psychotic experience through aberrant processing of salience.^36,37^ This failure to detect increased dopamine synthesis in nonclinical individuals with hallucinations suggests further differences at the neurobiological level which may be associated with the process of reality discrimination. This may in part explain the phenomenological differences in the experience of hallucinations in individuals with and without a clinical diagnosis.

Wider structural evidence also supports the association between morphological variation in the PCS and reality monitoring efficiency that underlies this model. The absence of a left hemisphere PCS in healthy individuals is associated with a reduction in GMV in paracingulate cortex, as well as an increase in volume in the surrounding ACC.^38^ Furthermore, Buda et al’s study (linking impaired reality monitoring ability in healthy individuals to the bilateral absence of the PCS^13^) showed an associated GMV increase in the mPFC. These results are consistent with the observation that reduced PCS cortical folding affects both local activation patterns,^39–41^ and cognitive functioning^14,42^ by altering the structural integrity of surrounding cortex. However, there may be a greater impact from reduced paracingulate folding on cognitive function due to weakened connectivity between the dorsal ACC and more distal brain regions, in particular, those involved in sensory processing such as speech-sensitive auditory cortex in the superior temporal gyrus. Such differences in connectivity might arise from factors related to the process of cortical folding that occurs during gestation.^43,44^ In this case, the PCS morphological differences we detect would simply be markers of the underlying cause of the associated cognitive variability. This too offers a focus for further research, with existing structural connectivity studies of hallucinations broadly supportive of such an explanation.^45,46^ Furthermore, Vercammen et al^47^ found evidence of reduced functional connectivity between the left temporoparietal junction and bilateral ACC associated with more severe AVHs in patients with schizophrenia.

The proposed model of hallucinations described is speculative, but it accounts for the broad similarity in the location of brain activity during hallucinations in clinical and nonclinical groups, particularly in the region of speech-sensitive auditory processing regions within the superior temporal gyrus.^30^ The model is also consistent with failures to detect reality-monitoring impairment in healthy individuals prone to hallucinations, as well as with the observation of spontaneous activity in speech sensitive auditory cortex in healthy individuals during periods of silence.^48^ While imaging studies of reality monitoring typically report mPFC activity in the frontal pole, this may relate particularly to declarative task-related activity, and more posterior/dorsal ACC activity is also observed in many of these studies (eg, refs.^8,9^). The proposed involvement of dorsal ACC in reality monitoring of sensory information is also consistent with a wider function of this region in error monitoring, attention, and the integration of cognitive and affective processes in executive control.^49–51^

The model can also accommodate the phenomenological differences often reported in the experience of clinical and nonclinical hallucinations. It is suggested that nonclinical hallucinations are unlikely to occur without hyper-activation of sensory cortices, which culminates in content which is unusually intense or vivid in nature. While a similar process is implicated for clinical hallucinations, this factor may be less significant, given the additional impact of impaired reality discrimination processes. Such an view can account for the lower frequency and duration of nonclinical, compared with clinical, hallucinations,^3,27^ as the experience of a nonclinical hallucination might depend on the sensory information surpassing, and being maintained at a level in excess of a hallucination threshold. Furthermore, nonclinical hallucinations are associated with a greater level of control than clinical hallucinations^3,27^ which might be understandable in terms of the relative ability to down-regulate sensory activity, rather than to enhance the impaired reality discrimination process intrinsic to clinical hallucinations. It is not, however, suggested that the model can account for the entirety of the experiential differences in hallucinations between the groups; for example, it cannot explain the enhanced level of negative content that may be associated with clinical hallucinations.^3,25,26^

Looking more broadly at the proposed model, there is also strong evidence supporting a role for ACC in the generation of hallucinations. Hunter et al’s^48^ demonstration of spontaneous activity in speech-sensitive auditory cortex in healthy individuals also found this to be associated with activity within the ACC. Dorsal ACC/paracingulate activity has also been related to the monitoring and generation of internal and external speech in healthy individuals and in patients with schizophrenia.^39,52^ ACC activity during hallucinations has been reported in some, but not all state studies of hallucinations (eg, Diederen et al^53^), in the generation of conditioned hallucinations in healthy individuals,^54^ and in self-induced hallucinations in hypnosis-prone individuals.^55^ Furthermore, a recent study into the processing of ambiguous speech in individuals with nonclinical hallucinations found that voice-hearers recognized the presence of speech in degraded sine-wave speech before control subjects, with the intelligibility response related to activity in both dorsal ACC and superior frontal gyrus.^56^ This suggests a role for ACC in the enhanced tendency of hallucination-prone individuals to extract meaningful linguistic content from ambiguous information.

This last finding is significant in highlighting the likely complexity of the hallucinatory process. Although our framework is admittedly simple, it might provide a useful basis to assist the understanding of hallucinations across clinical and nonclinical groups, and areas for future focus have been discussed above. The source monitoring framework^5^ suggests that decisions are made as to the source of a percept through comparison of its contextual, semantic, perceptual, or cognitive features with characteristic traces relating to internal or external sources.^57^ However, current computational theories of hallucinations propose instead a top-down driven process combining sensory information within a framework consisting of variable levels of beliefs and prior experience.^54,58^ It can be argued that these are not mutually exclusive processes—while the features of a percept may be generated through a process involving top-down and bottom-up interaction, it remains a failure in source attribution which underlies the experience of a hallucination as a false percept. The relative involvement of reality monitoring, attentional, and perceptual processes in the attribution of the source of sensory information remains an intriguing question, suggesting promising avenues for future research.

### Limitations

PCS length and local gyrification measures in the vicinity of the PCS for nonclinical individuals with hallucinations were intermediate between those for clinical individuals with hallucinations and control subjects without hallucinations. The failure to find a significant difference between nonclinical individuals with and without hallucinations, which would support a continuum model of hallucinations in clinical and nonclinical groups, may thus reflect an effect-size issue. Replication of this study or use of a larger dataset would address this, but confidence on this issue is obtained from the consistency of the findings between manual measures of PCS length, and brain-wide automated gyrification analyses involving nonparametric cluster-wise correction for multiple comparisons using Monte Carlo simulation. In both cases, no significant differences were found between nonclinical individuals with and without hallucinations. In contrast, significant differences in these measures were found between clinical individuals with hallucinations and nonclinical individuals without hallucinations (above), and between patients with schizophrenia with and without hallucinations in a previous study which utilized the same techniques.^15^ Further research is needed to assess the extent to which the present results generalize to other nonclinical populations with hallucinations who may have different etiology and phenomenology of hallucinatory experience.

In sum, we replicated earlier findings of shorter PCS in patients with hallucinations, but did not find this characteristic in nonclinical people with hallucinations. These findings, together with our previous work, corroborate a model in which part of the mechanism underlying hallucinations is shared between clinical and nonclinical individuals with hallucinations, while a second mechanism, compromising adequate reality monitoring, is present in patients only.

## Funding

J.R.G. and C.F. were supported by Wellcome Trust grants (WT098455 and WT108720). J.S.S. was supported by James S. McDonnell Foundation award (220020333). I.S. was supported by Vidi grant (017106301) from NWO.

## Supplementary Material

sby157_suppl_Supplementary_Material
