# Supplementary material for: Paracingulate Sulcus Morphology and Hallucinations in Clinical and Nonclinical Groups
Source: Schizophr Bull. 2018 Oct 30;45(4):733–41. doi: 10.1093/schbul/sby157 (PMC6581129; doi:10.1093/schbul/sby157)
Supplement: sby157_suppl_Supplementary_Material [file sby157_suppl_supplementary_material.doc]

# **Supplementary materials**

# **Recruitment Procedure**

The recruitment procedure for healthy control and non-clinical participants with AVHs has been described previously (e.g. 1). In brief, controls subjects and non-clinical individuals with AVHs were recruited via the www.verkenuwgeest.nl (‘Explore Your Mind’) website and invited for interview if they had AVH at least weekly (hallucinating group) or had not experienced AVH in their lifetime (control group). In a face-to-face interview the following inclusion criteria were checked: (i) participants had no current or past psychiatric disorders as assessed by the Comprehensive Assessment of Symptoms and History (CASH) interview and the Structured Clinical Interview for Diagnostic and Statistical Manual of Mental Disorders-III-R personality disorders (SCID-II) to exclude axis I and axis II pathology(2,3). Importantly, hallucinating subjects were free of delusions, disorganization, and negative symptoms. Anxiety or depressive disorder in full remission were not considered exclusion criteria. Additional exclusion criteria were (ii) no chronic somatic disorder (e.g., heart failure); (iii) absence of alcohol or drug abuse for at least 3 months before the assessments. Additional inclusion criteria for participants with AVH consisted of (iv) voices that were distinct from thoughts and had a ‘hearing’ quality; (v) voices that were experienced at least once a month; and (vi) drug or alcohol abuse did not precede the first experience of AVH. All patients with a psychotic disorder were recruited from the Psychiatry Department of the University Medical Centre Utrecht, The Netherlands. Patients were diagnosed using the CASH interview according to DSM-IV criteria by an independent psychiatrist(2).

# **Imaging Data**

T1-weighted structural MRI scans were obtained using a 3T Phillips scanner, using a 3D T1 turbo field-echo sequence (repetition time 9.96ms, echo time 4.59ms, flip angle 8°, field of view 224mm, matrix 256 mm x 256 mm, voxel size .875 x .875 x 1mm). Scan time was 8 mins 50 secs.

# **Measurement of PCS length**

The PCS measurement process has been described previously(4) and is openly available at <https://www.repository.cam.ac.uk/handle/1810/264520>). In essence, individual scans were imported as nifti folders into Mango brain visualization software (version 3.6; [http://ric.uthscsa.edu/mango)](http://ric.uthscsa.edu/mango/mango.html) and inspected for integrity. In an axial view, the locations of the anterior and posterior commissures (AC and PC) were marked, and the scan rotated to line up the AC and PC in a horizontal plane. The origin was reset to the location of the AC. On a sagittal slice, 4.375mm to the left or right of the medial line, the cingulate sulcus (CS) was identified as the first major sulcus running in an anterior–posterior direction, dorsal to the corpus callosum and typically visible for five sagittal slices or more. The PCS was then identified if present as a salient sulcus, running parallel, horizontal and dorsal to the CS, and visible for three or more sagittal slices measured from the medial plane (x = 0). The sulcus was measured using the ‘trace line’ function in Mango from its start in the first quadrant prescribed by y > 0 and the horizontal line linking the AC and PC (z > 0), starting at the point at which the sulcus ran in a posterior direction. The PCS was measured to its end point, which could fall outside of the first quadrant (Fig. 1).

# **Calculation of local gyrification index**

Measures of cortical folding across the brain were obtained by calculating local gyrification indices (lGI) for each MRI structural scan using the method of Schaer et al(5). This technique provides an automated measure of cortical folding by assigning a gyrification index value (broadly defined as the amount of the brain’s pial surface which is folded inwards in the sulci within a localised sphere) to thousands of points over the cortical surface(5). As lGI gives a measure of cortical curvature, it is a distinct morphological variable from sulcal length. However, our previous work has indicated that longer PCS length in a sample of patients with schizophrenia and healthy controls is moderately and positively correlated with mean lGI in surrounding regions(6). As such, lGI analysis provides a valuable method of automated validation for the manual tracing findings.

lGI gives a measure of cortical folding by comparing the amount of cortex buried within sulcal folds at the grey/white matter interface with the amount of visible or surface cortex at each vertex of the reconstructed brain surface, based on 3D spheres of radius 25 mm. Calculations made at each vertex can then be averaged to give a global gyrification index for each hemisphere, or for 34 individual brain regions as defined by an automated parcellation procedure(7). Calculation was undertaken using FreeSurfer Software version 5.3.0 ([http://surfer.nmr.mgh.harvard.edu](http://surfer.nmr.mgh.harvard.edu/)), which also provided measures of intracranial volume and cortical surface area for each subject’s scan, for use as covariates in the analysis. For group level whole brain statistical analysis, individual gyrification maps were registered to the Freesurfer average subject template and smoothed with a 5mm full-width, half maximum isotropic Gaussian kernel. Group differences in local gyrification were analysed by fitting a general linear model at each vertex on the surface. Group differences in lGI were analysed by fitting a general linear model at each vertex on the surface. Age was included as a covariate, and non-parametric cluster-wise correction for multiple comparisons was performed using Monte Carlo simulation (10,000 random permutations)(8), with a threshold of p < 0.05, corrected for multiple comparisons across each hemisphere.

A separate analysis was carried out using two-sample t-tests to identify region-specific differences averaged across *a priori* mPFC regions of interest in the vicinity of the PCS(7), at a threshold of p < 0.05. Regional differences outside the paracingulate region of interest, (taken to be bilateral frontopolar, medial orbitofrontal, superior frontal and paracentral parcellations from the Desikan atlas(7)), were reported if they exceeded a threshold of p < 0.05, corrected for multiple comparisons across the 34 parcellated brain regions.

1. Diederen KMJ, Daalman K, de Weijer AD, Neggers SFW, van Gastel W, Blom JD, et al. Auditory hallucinations elicit similar brain activation in psychotic and nonpsychotic individuals. Schizophrenia Bulletin. 2012;38(5):1074–82.

2. Andreasen NC, Flaum M, Arndt S. Comprehensive Assessment of Symptoms and History (CASH): An Instrument for Assessing Diagnosis and Psychopathology. Arch Gen Psychiatry. 1992;49:615–23.

3. First MB, Spitzer RL, Gibbon M, Williams JBW. Dsm-Iii-R Personality Disorders Part I : Description (Scid-Ii). Journal of Personality Disorders. 1995;9(2):83–91.

4. Garrison JR, Fernyhough C, McCarthy-Jones S, Haggard M, The Australian Schizophrenia Research Bank, Simons JS. Paracingulate sulcus morphology is associated with hallucinations in the human brain. Nature Communications. 2015;6:8956.

5. Schaer M, Cuadra MB, Tamarit L, Lazeyras F, Eliez S, Thiran J-P. A surface-based approach to quantify local cortical gyrification. IEEE transactions on medical imaging. 2008;27(2):161–70.

6. Garrison JR. Reality Monitoring and Hallucinations. PhD Thesis, University of Cambridge; 2015.

7. Desikan RS, Ségonne F, Fischl B, Quinn BT, Dickerson BC, Blacker D, et al. An automated labeling system for subdividing the human cerebral cortex on MRI scans into gyral based regions of interest. NeuroImage. 2006;31(3):968–80.

8. Hagler DJ, Saygin AP, Sereno MI. Smoothing and cluster thresholding for cortical surface-based group analysis of fMRI data. NeuroImage. 2006;33(4):1093–103.
